# A New **Subject**-**Specific** Discriminative and Multi-**Scale** Filter Bank Tangent Space Mapping Method for Recognition of Multiclass Motor **Imagery**

**DOI:** 10.3389/fnhum.2021.595723

**Published:** 2021-03-08

**Authors:** Fan Wu, Anmin Gong, Hongyun Li, Lei Zhao, Wei Zhang, Yunfa Fu

**Affiliations:** ^1^Faculty of Information Engineering and Automation, Kunming University of Science and Technology, Kunming, China; ^2^Brain Cognition and Brain-Computer Intelligence Fusion Innovation Group, Kunming University of Science and Technology, Kunming, China; ^3^Department of Information Engineering, Engineering University of PAP, Xi’an, China; ^4^College of Science, Kunming University of Science and Technology, Kunming, China; ^5^Kunming Medical University, Kunming, China; ^6^Yunnan Provincial Key Laboratory of Computer Technology Application, Kunming, China; ^7^School of Medicine, Kunming University of Science and Technology, Kunming, China

**Keywords:** tangent space mapping, discriminative and multiscale filter bank, multiclass motor-imagery, Riemannian geometry based classifier, electroencephalogram

## Abstract

**Objective:** Tangent Space Mapping (TSM) using the geometric structure of the covariance matrices is an effective method to recognize multiclass motor imagery (MI). Compared with the traditional CSP method, the Riemann geometric method based on TSM takes into account the nonlinear information contained in the covariance matrix, and can extract more abundant and effective features. Moreover, the method is an unsupervised operation, which can reduce the time of feature extraction. However, EEG features induced by MI mental activities of different subjects are not the same, so selection of subject-specific discriminative EEG frequency components play a vital role in the recognition of multiclass MI. In order to solve the problem, a discriminative and multi-scale filter bank tangent space mapping (DMFBTSM) algorithm is proposed in this article to design the subject-specific Filter Bank (FB) so as to effectively recognize multiclass MI tasks.

**Methods:** On the 4-class BCI competition IV-2a dataset, first, a non-parametric method of multivariate analysis of variance (MANOVA) based on the sum of squared distances is used to select discriminative frequency bands for a subject; next, a multi-scale FB is generated according to the range of these frequency bands, and then decompose multi-channel EEG of the subject into multiple sub-bands combined with several time windows. Then TSM algorithm is used to estimate Riemannian tangent space features in each sub-band and finally a liner Support Vector Machines (SVM) is used for classification.

**Main Results:** The analysis results show that the proposed discriminative FB enhances the multi-scale TSM algorithm, improves the classification accuracy and reduces the execution time during training and testing. On the 4-class BCI competition IV-2a dataset, the average session to session classification accuracy of nine subjects reached 77.33 ± 12.3%. When the training time and the test time are similar, the average classification accuracy is 2.56% higher than the latest TSM method based on multi-scale filter bank analysis technology. When the classification accuracy is similar, the training speed is increased by more than three times, and the test speed is increased two times more. Compared with Supervised Fisher Geodesic Minimum Distance to the Mean (Supervised FGMDRM), another new variant based on Riemann geometry classifier, the average accuracy is 3.36% higher, we also compared with the latest Deep Learning method, and the average accuracy of 10-fold cross validation improved by 2.58%.

**Conclusion:** Research shows that the proposed DMFBTSM algorithm can improve the classification accuracy of MI tasks.

**Significance:** Compared with the MFBTSM algorithm, the algorithm proposed in this article is expected to select frequency bands with good separability for specific subject to improve the classification accuracy of multiclass MI tasks and reduce the feature dimension to reduce training time and testing time.

## Introduction

Brain-computer interface (BCI) is a revolutionizing human-computer interaction (Graimann et al., 2010), and BCI based on motor imagery (MI-BCI) is an important type of BCI which is expected to provide communication and control with the outside world for patients with severe motor disabilities (Wolpaw and Wolpaw, 2012), especially in motor dysfunction rehabilitation training (Soares et al., 2013). However, at present, MI-BCI can classify few MI tasks, and it can provide few effective instructions, which limits the communication capability and control freedom of this type of BCI, making it difficult to enter practical applications. In order to add instructions, it is necessary to study the recognition of multiclass MI tasks. At present, the recognition accuracy of multi-class MI needs to be improved, which is a challenging work. This article intends to explore effective methods to improve the recognition accuracy of multi-class MI.

Neuroscience research has shown that brain activities related to MI and motor execution (ME) can cause similar sensorimotor rhythm changes (Pfurtscheller and Neuper, 1997), and the EEG amplitude of certain frequency bands will decrease event-related desynchronization (ERD) or increase event related synchronization (ERS). This ERD/ERS phenomenon or pattern is most prominent in mu rhythm (8–12 Hz) and beta rhythm (13–30 Hz), and can also be observed in gamma rhythm close to 40 Hz (Rao, 2013). In MI-BCI, these patterns are mainly extracted. However, due to the non-stationarity of EEG, low signal-to-noise ratio and limited available calibration data, it is difficult to extract MI feature patterns with good separability (Lotte et al., 2018). In MI-BCI, the classical processing method is to extract sources from the pre-processed EEG data using a spatial filter such as CSP, then extract the feature vectors from the source signal, and finally classify the feature vectors using a vector-based classifier (such as LDA) (Yger et al., 2017). Studies have shown that Common Spatial Pattern (CSP) has significant advantages in extracting MI features (Lotte et al., 2018)CSP maximizes the variance of the EEG signal of one class of MI while minimizing the variance of the other class. After band-pass filtering, the variance of the EEG signal is the power of the corresponding frequency band. Therefore, CSP is a more suitable method to extract the features of the two classes of MI (Ramoser et al., 2000). Deep Learning is a specific machine learning algorithm in which features and the classifier are jointly learned directly from data (Lotte et al., 2018). Advantages of Deep Learning include that they are well suited for end-to-end learning, that is, learning from the raw data without any *a priori* feature selection, that they scale well to large datasets, and that they can exploit hierarchical structure in natural signals (Schirrmeister et al., 2017). Disadvantages of Deep Learning methods include that they may output false predictions with high confidence may require a large amount of training data, may take longer to train than simpler models, and involve a large number of hyperparameters such as the number of layers or the type of activation function (Nguyen et al., 2015). Convolutional neural networks (ConvNets) are the most popular Deep Learning approaches for BCI (Lotte et al., 2018). In order to adapt the existing ConvNets architectures from the field of computer vision to EEG input, the authors created three ConvNets with different architectures, with the number of convolutional layers ranging from 2 layers in a “shallow” ConvNet over a 5-layer deep ConvNet up to a 31-layer residual network (ResNet) (Schirrmeister et al., 2017). In Sakhavi et al. (2018), according to the features generated by filter bank CSP (FBCSP), the authors design and optimize a ConvNet for classification.

In addition to CSP and its various improvement methods (Ang et al., 2008, 2012; Zhang et al., 2015, 2016), the researchers used the Riemannian method based on the covariance matrix in the Riemannian manifold in MI-BCI and achieved better performance, and this new processing method does not require source extraction. At present, Riemannian manifold of symmetric positive definite (SPD) matrices has attracted more and more attention due to their rich framework for manipulating the covariance structure of the data. The concept of the covariance matrices in the manifold has been successfully used in radar signal processing (Barbaresco, 2008), diffusion tensor Imaging (Fletcher and Joshi, 2004) and computer vision (Tuzel et al., 2008). A similar method is combined with K nearest neighbors and recognizes different sleep states based on EEG (Li et al., 2009). Barachant et al. (2010) first used the Riemannian method to classify two-class MI-EEG data and achieved an average classification accuracy of 85.2%. The Minimum Distance to Riemannian Mean (MDRM) introduced in their works is the most basic Riemannian method (Congedo et al., 2017). In this method, the Riemannian mean of each class is calculated first based on the training data, and then classify incoming trials by comparing the Riemannian distances between the covariance matrices corresponding to the incoming trials and the Riemannian mean of each class during the test session (Barachant et al., 2010). Another more sophisticated and effective Riemannian classifiers is based on tangent space mapping (TSM), and its classification performance is significantly better than CSP and other methods (Congedo et al., 2017). Barachant et al. mapped the covariance matrices onto the tangent space, and then selected features in it and used LDA, the results showed that compared with MDRM, it can significantly improve the accuracy of multi-class (4-class) MI recognition (Barachant et al., 2012). Barachant et al. (2013) derived a new kernel by establishing a connection with the Riemannian geometry of symmetric positive definite matrices, and combined with a support vector machine to test different kernels, and demonstrated that this new approach outperformed significantly state of the art results, effectively replacing the traditional spatial filtering approach.

In order to further improve the classification performance of MI-BCI, Ang et al. (2008) proposed the filter bank CSP (FBCSP) method, a four-stage procedure in which CSP is applied at several fixed frequency bands, and where the most relevant sub-band CSP features are automatically pair-wise selected based upon mutual information criteria. Recently, Zhang et al. (2015) proposed the sparse filter bank CSP (SFBCSP) in which a small number of sub-band CSP features are automatically selected based on LASSO (least absolute shrinkage and selection operator) regression. According to some recent achievements, we know that a breakthrough has been made in the research of MI task recognition based on Deep Learning (Li et al., 2019; Olivas Padilla and Chacon Murguia, 2019; Xu et al., 2020). In Xu et al. (2020), a new deep multi-view feature learning method for the classification task of motor imagery electroencephalogram (EEG) signals is proposed in order to obtain more representative motor imagery features in EEG signals. In Li et al. (2019), the researchers proposes a variant of Discriminative Filter Bank Common Spatial Pattern (DFBCSP) for extracting MI features, and then sets the resulting samples into a matrix, which is then fed to one or many ConvNets previously optimized by using a Bayesian optimization for classification. In Olivas Padilla and Chacon Murguia (2019), a densely feature fusion convolutional neural networks (DFFN) is proposed. DFFN takes into account the correlation between adjacent layers and cross-layer features, thus reducing information loss in the process of convolutional operation. It also takes into account the local and global characteristics of the network, and improves the identification accuracy of the ordinary ConvNets framework in multi-class MI. In the improvement of the method based on Riemannian geometry, Barachant et al. proposed Fisher Geodesic Discriminant Analysis for performing Geodesic filtering to make the classes more separable along the geodesics, which improves the drawback of MDRM not taking into account intra-class distribution (Barachant et al., 2010). More recently, Satyam et al., combined the two adaptive strategies of RETRAIN and REBIAS (Shenoy et al., 2006) with MRDM and Fisher Geodesic Minimum Distance to Riemannian Mean (FgMDRM), and the result achieved an average classification accuracy of approximately 74% on the test set (Session 2) of the 2a data set of BCI Competition IV (Kumar et al., 2019). Islam et al. (2017) proposed a multi-band TSM method, which takes into account multiple frequency bands and helps to extract effective noise robust features for narrow-band signals, but the study did not consider the question of the subject-specific frequency band. However, MI-BCI is an active BCI. The EEG features induced by MI mental activity of different subjects are often different. It is necessary to customize the feature extraction method for specific subjects. Islam et al. proposed a multiband tangent space mapping with sub-band selection (MTSMS). The sub-band selection method adopted can be based on the mutual information between features and class labels, thereby effectively extract the frequency band of a specific subject, and further improve the performance of MI-BCI (Islam et al., 2018). In addition, in order to overcome the limitation of using fixed band window analysis in MI-BCI, Hersche et al. (2018) proposed a multi-scale filter bank TSM (MFBTSM), in which FB contains the frequency bands are multi-scale and overlapping. At the same time, multi-scale and overlapping time windows are divided, so that multiple time windows are used to analyze EEG trials and perform FB analysis in each time window. This greatly increases the number of tangent spatial features, but induce redundant information. The disadvantages of MFBTSM is that the filter bank used by each subject is the same, and the test time and training time increase due to the large feature dimension.

In order to make up for the disadvantages of MFBTSM, this article intends to use a non-parametric method of multivariate analysis of variance based on the sum of squared distances to select the subject-specific discriminative EEG frequency components, and these component is vital for identifying multiple types of MI tasks. It is important to use multi-scale filter bank TSM at the same time, and finally use SVM for classification.

## Materials and Methods

### Riemann Geometry Associated With the Proposed Method

#### EEG Signals Are Represented as Covariance Matrices

To use Riemannian geometry to process EEG signals, it is necessary to represent the EEG signals as covariance matrices, which are SPD matrices. Let X*i* ∈ R^*N*_*c*_ × *N*_*s*_^ be the MI EEG signal of the *i*-th trial, where *N*_*c*_ is the number of channels and *N*_*s*_ is the number of samples. The sample covariance matrix (SCM) of the *i*-th trial is denoted by *P*_*i*_ ∈ R^*N*_*c*_ × *N*_*c*_^, which is estimated by eq. (1) (Barachant et al., 2012):

(1) $$P_{i}=1/\left(N_{s}-1\right)\,X_{i}\,X_{i}^{T}$$

Let S(n) denote the set of *n*×*n* symmetric matrices, and *P*(*n*) denote the set of *n*×*n* SPD matrices.

#### Riemannian Manifold and Tangent Space

The space of SPD matrices *P*(*n*) is a differentiable Riemannian manifold ℳ (Förstner and Moonen, 2003). The derivatives at a matrix **P** on the manifold lies in a vector space *T*_***P***_, which is the tangent space at that point. The tangent space is lying in the space *S*(*n*). The manifold and the tangent space are m  =  n(n++1)/2 dimensional.

Each tangent space has an inner product ⟨,⟩*P* that varies smoothly from point to point over the manifold. The natural metric on the manifold of SPD matrices is defined by the local inner product:

(2) $${\left\langle {\text{S}}_{1},{\text{S}}_{2}\right\rangle }_{P}=\text{Tr}\,\left({\text{S}}_{1}\,{\text{P}}^{-1}\,{\text{S}}_{2}\,{\text{P}}^{-1}\right)$$

The inner product induces a norm for the tangent vectors on the tangent space, such that, ${∥S∥}_{P}^{2}={\left\langle S,S\right\rangle }_{P}=\mathrm{Tr}\,\left({\text{SP}}^{-1}\,{\text{SP}}^{-1}\right)$. We note that, at Identity matrix, such norm simplifies into the Frobenius norm, i.e., ${\left\langle S,S\right\rangle }_{I}=∥S∥{}_{F}^{2}$.

#### Riemannian Geodesic Distance and Riemannian Distance

Let **Γ**(*t*) : [0,1]→**P**(*n*) be any (differentiable) path from Γ (0) = **P**_1_ to Γ (1) = **P**_2_. The length of Γ (*t*) is given by:

(3) $$L\,\left(\Gamma \,\left(t\right)\right)=\int_{0}^{1}{∥\dot{\Gamma }\,\left(t\right)∥}_{\Gamma \,\left(t\right)}\,dt$$

With the norm defined previously. The minimum length curve connecting two points on the manifold is called the geodesic, and the Riemannian distance between the two points is given by the length of this curve. The natural metric (2) induces the geodesic distance (Moakher, 2005):

(4) $$\delta_{R}\left({\text{P}}_{1},{\text{P}}_{2}\right)=∥\text{log}\left({\text{P}}_{1}^{-1}\text{P}{}_{2}\right){∥}_{F}={\left[\sum_{i=1}^{n}{\text{log}}^{2}\lambda_{i}\right]}^{1/2}$$

Where, λ_*i*_,*i* = 1…*n* are the real eigenvalues of ${\text{P}}_{1}^{-1}\,{\text{P}}_{2}$.

#### Exponential Map

For each point **P** ∈ *P*(*n*), we can thus define a tangent space composed by the set of tangent vectors at **P**. Each tangent vector **S**_*i*_ can be seen as the derivative at *t* = 0 of the geodesic Γ_*i*_(*t*) between **P** and the exponential mapping **P**_*i*_ = Exp_**P**_(**S**_*i*_), defined as:

(5) $${\text{Exp}}_{P}\,\left({\text{S}}_{i}\right)={\text{P}}_{i}={\text{P}}^{\frac{1}{2}}\,\text{exp}\,\left({\text{P}}^{-\frac{1}{2}}\,{\text{S}}_{i}\,{\text{P}}^{-\frac{1}{2}}\right)\,{\text{P}}^{\frac{1}{2}}$$

The inverse mapping is given by the logarithmic mapping defined as:

(6) $${\text{log}}_{P}\,\left({\text{P}}_{i}\right)={\text{S}}_{i}={\text{P}}^{\frac{1}{2}}\,\text{log}\,\left({\text{P}}^{-\frac{1}{2}}\,{\text{P}}_{i}\,{\text{P}}^{-\frac{1}{2}}\right)\,{\text{P}}^{\frac{1}{2}}$$

#### Euclidean Mean

Using the Euclidean distance on ℳ(*n*),δ_*E*_(**P**_1_,**P**_2_) = ∥ ***P***_1_−***P***_2_ ∥ *F*, it is possible to define the Euclidean mean of *I*≥ 1SPD matrices by:

(7) $$𝔄\,\left({\text{P}}_{1},\ldots ,{\text{P}}_{I}\right)=\underset{P\in P\,\left(n\right)}{\arg m\,i\,n}\sum_{i=1}^{I}\delta_{E}^{2}\,\left(P,{\text{P}}_{i}\right)=\frac{1}{I}\,\sum_{i=1}^{I}{\text{P}}_{i}$$

#### Riemannian Mean

Similar to Euclidean mean, Karcher/Fréchet means extends the notion of mean/center of mass to *P*(*n*) by estimating the SPD matrix which minimizes the sum of squared AIRM distances to all the SPD matrices in the set. Mathematically the Riemannian mean of *I* ≥ 1SPD matrices is given by:

(8) $$𝔊\,\left({\text{P}}_{1},\ldots ,{\text{P}}_{I}\right)=\underset{P\in P\,\left(n\right)}{\arg m\,i\,n}\sum_{i=1}^{I}\delta_{R}^{2}\,\left(P,{\text{P}}_{i}\right)$$

Eq. (8) has a unique minimum, and there is no closed solution for *I* >  2, but many iterative algorithms solve this problem through numerical analysis (Moakher, 2005).

### Discriminative and Multi-Scale Filter Bank Tangent Space Mapping

**FIGURE 1:**
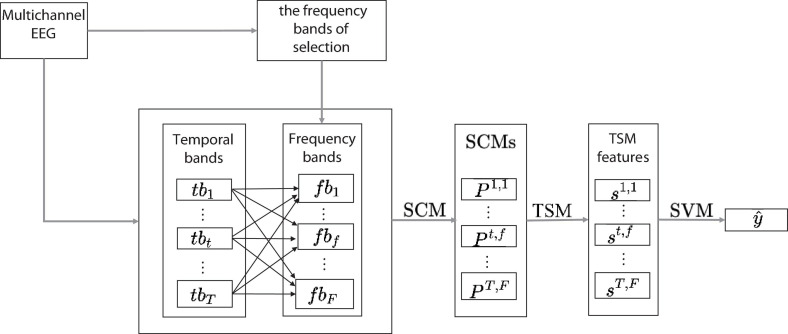
DMFBTSM structure diagram. After generating DMFB, first decompose the multi-channel EEG signal into T temporal windows (*tb*_1_,…,*tb*_*t*_,…,*tb*_*T*_), then use DMFB to decompose the *t*-th temporal window into *F* (*fb*_1_,…,*fb*_*f*_…,*fb*_*F*_) frequency bands, estimate the SCM in each time band, and then use the TSM algorithm to map all SCMs onto the tangent space to extract TSM features, and finally use linear SVM for classification.

The structure of Discriminative and Multi-scale Filter Bank Tangent Space Mapping (DMFBTSM) proposed in this article is shown in Figure 1. First, a set of filters is used to decompose the multi-channel EEG signal into multiple frequency band components. These filters are called the parent filter bank (Filter Bank, FB), and the parent FB covers all frequency components in the range of 2–40 Hz. Then use the one-way multivariate analysis of variance (MANOVA) based on the sum of squared distances to calculate the F statistic for each sub-band component decomposed. According to the F statistic, select EEG frequency bands that are separable for MI of the specific subject, and then generate discriminative and multi-scale filter bank (DMFB).

#### The One-Way MANOVA Based on the Sum of Squared Distances

In this article, a non-parametric method of MANOVA based on the sum of squared distances (Anderson, 2001) is used to select the EEG frequency bands that are separable for MI of the specific subject. The test statistic is a multivariate analog to Fisher’s F-ratio and is calculated directly from any symmetric distance or dissimilarity matrix.

**FIGURE 2:**
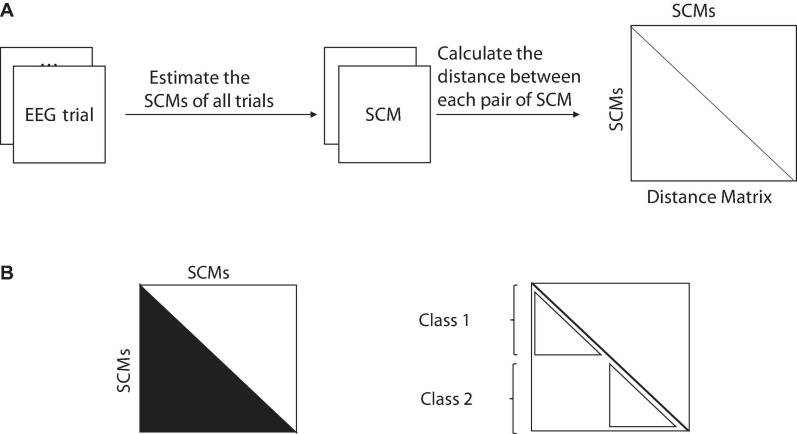
**(A)** From the raw data to the distance matrix and **(B)** a non-parametric MANOVA statistic for a one-way design (two groups) directly from the distance matrix. Sum of squared distances in the half matrix (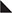) divided by N (the total number of SCMs in all classes) is the total sum of squares (*SS*_*T*_), and the sum of squared distances within classes (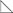) divided by n (number of SCMs in each class) is within-group sum of squares (*SS*_*W*_).

First, the EEG signals of a specific subject’s frequency range of 2–40 Hz are decomposed into 2 Hz width, a total of 19 sub-bands. Then estimate the SCMs of all trials in each sub-band and calculate the distance matrix between each pair of SCMs, as shown in Figure 2. Finally, the F statistic of each sub-band is calculated by MANOVA based on the square of the distance. The calculation process is as follows.

Assuming that the test data of the subject has a classes, each class has n trials, the total number of trials is *N* = a^∗^n, and the total sum of squares is:

(9) $$S\,S_{T}=\frac{1}{N}\,\sum_{i=1}^{N-1}\sum_{j=i+1}^{N}d_{i\,j}^{2}$$

where, *d*_*ij*_ is the distance between the SCM of the *i*-th trial and the SCM of the *j*-th trial. In a similar fashion, the within-group or residual sum of squares is:

(10) $$S\,S_{W}=\frac{1}{n}\,\sum_{i=1}^{N-1}\sum_{j=i+1}^{N}d_{i\,j}^{2}\,\epsilon_{i\,j}$$

where, if the i-th trial and the *j*-th trial are in the same class, the value of ϵ_*ij*_ is 1, otherwise it is 0, as shown in Figure 2B. The sum of squares between classes, *SS*_*A*_ and F statistics are calculated by eqs. (11, 12):

(11) $$S\,S_{A}=S\,S_{T}-S\,S_{W}$$

(12) $$F=\frac{S\,S_{A}/\left(a-1\right)}{S\,S_{W}/\left(N-a\right)}$$

In this article, the aforementioned Riemannian distance and Euclidean distance are applied to eqs. (9–12), respectively. If the sample points of different classes have different center positions in the multivariate space (centroid in the case of Euclidean distance), the ratio of the inter-class distance to the intra-class distance will be large, and the generated F-statistic will be relatively large. After calculating the F statistics of all sub-bands, arrange the sub-bands in descending order of F scores, take the first several separable sub-bands, and merge the adjacent separable sub-bands to obtain the EEG frequency bands that are separable for MI of the specific subject.

#### Divide Multi-Channel EEG Using Multi-Scale Time and Frequency Windows

**FIGURE 3:**
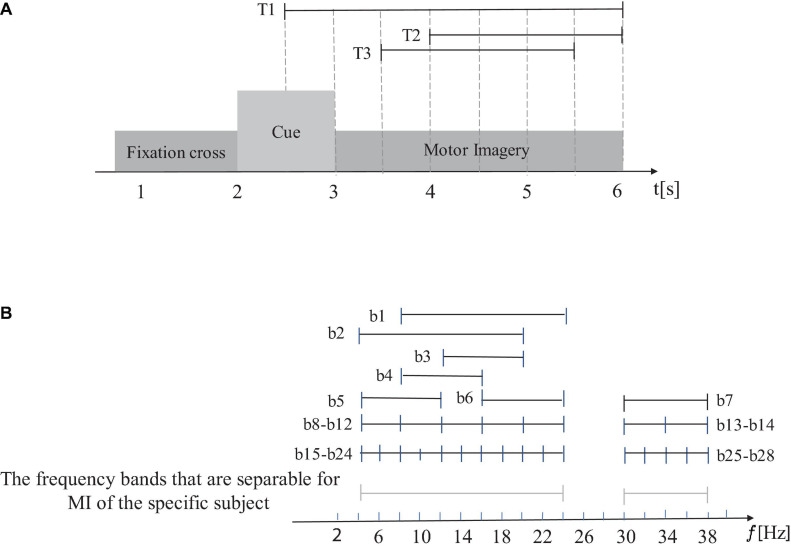
Divide multi-channel EEG using multi-scale time (temporal) and frequency windows. **(A)** The multi-channel EEG of a trial is divided according to the multi-scale time window. **(B)** The frequency bands that are separable for MI of the specific subject are divided according to the multi-scale frequency band windows.

First, the multi-channel EEG of a trial is divided according to the multi-scale time window shown in Figure 3A, and then according to the multi-scale frequency band window division shown in Figure 3B, the frequency bands that are separable for MI of the specific subject are divided according to the multi-scale frequency band windows shown in Figure 3B to generate DMFB, and then the DMFB band-pass filters the signal of each time window.

#### Tangent Space Mapping

**FIGURE 4:**
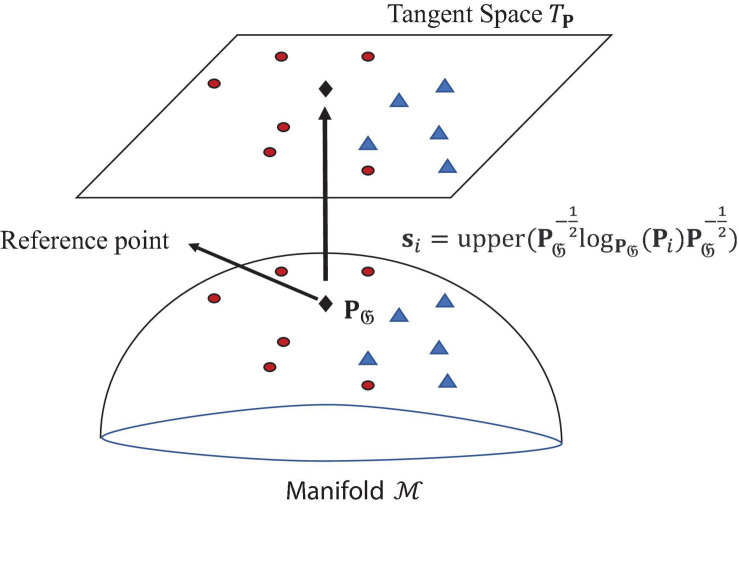
Schematic diagram of TSM on Riemannian manifold ℳ.

This article uses the TSM algorithm proposed by Barachant et al. (2010), as shown in Figure 4. The algorithm first needs to find a reference point **P**_*𝔊*_, which is the Riemann average of all EEG trials on manifold ℳ: **P**_*𝔊*_ = *𝔊*(**P**_*i*_,*i*  1…*I*). Then map the SCM corresponding to each trial onto the tangent space *T*_***P***_ to generate a set of *m* = *N*_*C*_(*N*_*C*_ + 1)/2-dimensional tangent vectors *S*[*s*_1_…*s*_*I*_] ∈ R*m* × *I*, The tangent vector **s**_*i*_ is calculated as eq. (13):

(13) $${\text{s}}_{i}=\text{upper}\,\left({\text{P}}_{𝔊}^{-\frac{1}{2}}\,{\text{log}}_{P_{𝔊}}\,\left({\text{P}}_{i}\right)\,{\text{P}}_{𝔊}^{-\frac{1}{2}}\right)$$

where, **P**_*i*_ is the SCM corresponding to the i-th trial, upper means to vectorize the upper triangular part of a SPD matrix, with appropriate weighting.

## Results

### Description of Data

First, analyze the justifiability of selecting frequency bands for specific subjects based on F statistics, using BCI Competition III dataset IVa and BCI competition IV dataset 2a^1^, and finally using BCI Competition IV 2a data set evaluation the performance of the proposed method.

#### BCI Competition IV Dataset 2a

Dataset 2a (Naeem et al., 2006) contains EEG data from 9 subjects who perform four kinds of motor imagery (right hand, left hand, foot, and tongue imagined movements). This dataset is provided by the Knowledge Discovery Institute (BCI Laboratory) of Graz University of Technology, Austria. EEG signals are recorded using 22 electrodes. For each subject, a training set (session 1) and a test set (session 2) are available. The same number of trials for all the MI tasks were provided for testing and training session. Each of the session had 72 trials for each of the four motor imagery classes.

#### BCI Competition III Dataset IVa

Dataset IVa (Dornhege et al., 2004) contains 2-class of MI EEG. This dataset is provided by the Knowledge Discovery Institute (BCI Laboratory) of Graz University of Technology, Austria. It records the EEG of 5 healthy subjects who perform two classes of MI (right hand and foot), Each subject recorded 280 trials, of which the first 168, 224, 84, 56, and 28 trials constituted the training set of subjects A1, A2, A3, A4, and A5, and the remaining trials constituted their test set.

### Experimental Results

#### F Statistic Selects the Frequency Bands That Are Separable for MI of the Specific Subject

**FIGURE 5:**
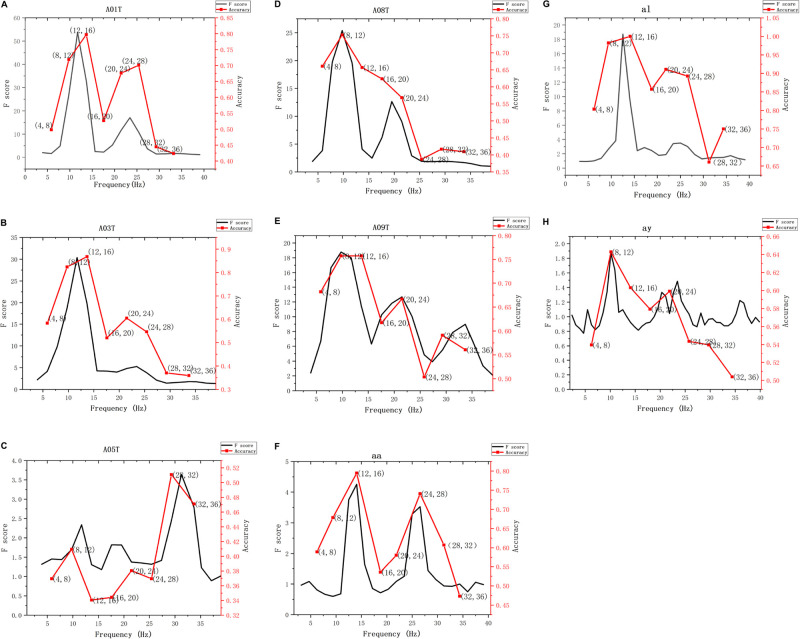
The F score and classification accuracy vary with the subjects and different frequency bands. **(A–H)** The F scores and classification accuracy of subjects A01T, A03T, A05T, A08T, A09T, aa, al, and ay vary with different frequency bands. The first five subjects are from data set IV- 2a, the last 3 subjects are from data set III-IVa.

Using the parent FB in the frequency range of 2 to 40 Hz, the EEG signal of each subject was decomposed into 19 sub-bands, and then the Riemannian distance was selected as the distance metric to calculate the F score of each sub-band. In order to show the justifiability of using the F score of each sub-band as the criterion for selecting a separable frequency band, the classification accuracy of different sub-bands of the test data of different subjects on the BCI competition public data set is calculated, as shown in Figure 5, where the sub-band width for calculating the classification accuracy is 4 Hz, and the range is from 4 to 36 Hz. It can be seen from Figure 5 that the classification accuracy of the sub-band with a higher F score is better than that of the sub-band with a lower F score. Therefore, it is justified to use one-way MANOVA based on the square of the distance to select the separable sub-bands. Then, the sub-bands are sorted in descending order of F score, and the top G sub-bands are used for MI classification.

#### Multi-Class MI (4-Class) Classification Results

In this study, nine subjects in the BCI competition IV data set 2a (four types of MI) were selected for separable frequency bands, and multi-scale time-frequency TSM features were extracted and classified. In order to better evaluate the performance of DMFBTSM, first compare with MFBTSM, the results are shown in Table 1, and then test other three related methods on the same data set. The first method is the combination of FgMDRM and RETAIN Adaptive strategy, called Supervised Adaptive FgMDRM (Supervised FgMDRM). In this method, the FgMDRM classifier is first trained on training/calibration session data, then during the testing session, the classifier is retrained after each prediction (Kumar et al., 2019). The second method is the combination of TSM and adaptive Riemannian kernel SVM, known as adaptive Riemannian kernel SVM (ARK-SVM) (Barachant et al., 2013), and the third method is FBCSP (Ang et al., 2012). Comparison results of these three methods with DMFBTSM are shown in Table 2.

**TABLE 1:** Under different distance measures [N/A (no distance metric), Euclidian distance and Riemannian distance], multi-scale time-frequency TSM features were extracted, and average classification accuracy (%) and standard deviation (std), average training time and average test time were obtained from the 4 MI classes on the test data (session 2) of 9 subjects in BCI Competition IV data-set 2a.

**Method**	**MFBTSM**		**DMFBTSM**			
Distance metric	N/A	N/A	Euclidian distance	Euclidian distance	Riemannian distance	Riemannian distance
Time window selection	T1	T1, T2, T5	T1	T1, T2, T5	T1	T1, T2, T5
A1	91.81	90.04	91.81	92.53	92.53	93.24
A2	51.59	55.48	56.54	61.13	55.48	60.78
A3	83.52	81.32	82.42	83.15	87.18	87.18
**A4**	73.25	71.92	69.74	70.18	70.18	71.49
A5	63.41	69.57	67.75	68.12	68.12	66.67
A6	58.60	56.74	61.4	59.53	60.0	61.4
A7	86.64	85.56	83.03	85.92	87.0	86.28
A8	81.55	83.76	80.81	83.39	83.39	85.61
A9	82.58	84.85	82.2	84.85	85.23	83.33
Mean	74.77	75.47	75.08	76.53	76.57	77.33
Std	13.9	12.8	11.7	12.0	13.4	12.3
Avg. training time [s]	34.32	55.39	10.43	29.78	11.04	32.68
Avg. testing time [s]	10.91	20.92	4.47	12.56	4.70	12.24

**TABLE 2:** Mean classification accuracy (%) and standard deviation (std) obtained across nine subjects in data-set 2a.

**Method**	**DMFBTSM**	**Supervised FgMDRM**	**FgMDRM**	**ARK-SVM**	**FBCSP**
Mean	77.33	73.97	68.31	65.29	67.21
Std	12.3	13.1	14.2	14.4	19.2

In addition, this article is compared with the latest three Deep Learning-based methods. In Deep Multi-view feature learning method (Xu et al., 2020), the author uses the improved, the deep restricted Boltzmann machine (RBM) network to learn to learn the multi-view features of EEG signals, and finally uses SVM to classify deep multi-view features. The DFFN algorithm is a dense feature fusion convolutional neural network using CSP and ConvNet technology (Li et al., 2019). In the Monolithic Network method (Olivas Padilla and Chacon Murguia, 2019), the authors used a variant of discriminative FBCSP to extract signal features, and then developed a Bayes-optimized ConvNet network for classification. The Shallow-ConvNet algorithm inspired by the FBCSP pipeline, specifically tailored to decode band power features (Schirrmeister et al., 2017). After extracting the FBCSP features, the CW-ConvNets algorithm inputs them into the ConvNets for classification (Sakhavi et al., 2018). Comparison results of the method proposed in this article and the three Deep Learning methods are shown in Table 3.

**TABLE 3:** Mean classification accuracy (%) and standard deviation (std) on test data by 10 fold cross-validation achieved by DMFBTSM, Deep Multi-View Feature Learning, Shallow-ConvNet, CW-ConvNet, Monolithic Network, and DFFN, for dataset 2a.

**Method**	**DMFBTSM**	**Deep Multi-View Feature Learning**	**Shallow-ConvNet**	**CW-ConvNet**	**Monolithic Network**	**DFFN**
Mean	81.0876	78.5074	71.86	73.07	78.41	76.44
Std	11.2	12.0	12.4	15.1	6.3	11.6

Tables 1, 2 present the mean and standard deviation of the classification accuracy (averaged across all the subjects) on a session to session transfer evaluation for these methods. The results presented in Table 3 are obtained by combining and randomly arranging the training data (Session 1) and test data (Session 2) of each subject’s data set according to the data organization method in Xu et al. (2020), and then performing 10 fold cross-validation.

In order to calculate the sub-band F score, Riemannian distance and Euclidean distance are selected and compared in this study. In addition, due to the differences in MI of different subjects, in order to ensure the accuracy of MI classification, the number of sub-bands G selected by each specific subject may not be the same. In addition, in order to ensure the accuracy of MI classification, the number of sub-bands G selected by each specific subject may not be the same. At the same time, in order to reduce the number of features to reduce training time and test time, the value of G ranges from 11 to 14. Specifically, subject 1 and 9 chose G as 13, subject 2, subject 3, subject 6, and subject 8 chose G as 11, subject 4 and 7 chose G as 14, and subject 5 chose G as 12. Choose one (T1) or three (T1, T2, and T3) time windows for decomposing EEG signals for comparison. In the case of one time window, the feature dimension of the subjects is 10879, and the feature dimension varies from 5060 to 7840 after frequency band selection. In addition, 10-fold cross-validation was used for the selection of time window and frequency band, as well as the determination of the SVM’s hyperparameter C.

In order to evaluate the computational cost of the proposed method, the average training and testing time of all trials for each subject is measured. The training time includes the preprocessing and training time of the classifier, and the testing time includes the feature extraction and classification time. The experiments were conducted on an Intel Core i5-7200U 2.71 GHz processor with 8 GB RAM.

Table 1 shows that the proposed discriminative FB enhances the multi-scale TSM algorithm. The best classification accuracy obtained by using Euclidean distance as the distance metric is 76.53 ± 12.0%, the shortest training time is 10.43 s, and the shortest test time is 4.47 s; The best classification accuracy obtained by using Riemannian distance as the distance metric is 77.33 ± 12.3%, the shortest training time is 11.04 s, and the shortest test time is 4.70 s.

## Discussion

Existing studies have shown that, compared with the conventional CSP method, Riemannian geometry based methods can bypass the spatial filtering of electrodes to make the calibration phase easier, and significantly improve the recognition accuracy of MI tasks (Barachant et al., 2012, 2013). In fact, the improvement brought by Riemannian geometry is due to the consideration of the non-linear information contained in the covariance matrices, thus better extracting features, which are usually discarded by the linear space filtering methods. On the basis, the multi-band Riemannian method can use a small amount of calibration data to extract the noise robust features, and achieve better results (Islam et al., 2017, 2018; Hersche et al., 2018). In order to further improve the multi-band Riemannian method, this article uses a non-parametric method of MANOVA based on the sum of squared distances (Anderson, 2001) to select frequency bands that are separable for specific subjects, and multi-scale division is performed on the multi-channel EEG signals in these frequency bands. Finally, use TSM to extract tangent space features.

It can be seen from Table 1 that when a time window (T1) is used, the classification accuracy of DMFBTSM using Euclidean distance is 0.31% higher than that of MFBTSM, the training time is shortened by more than three times, and the test time is shortened by more than two times; the classification accuracy of DMFBTSM using Riemannian distance is 1.8% higher than that of MFBTSM, the training time is shortened by more than three times, and the test time is shortened by more than two times. In the case of using three time windows (T1, T2, and T3), the classification accuracy of DMFBTSM using Euclidean distance is 1.06% higher than that of MFBTSM, training time is shortened by 1.9 times, and test time is shortened by 1.7 times; the classification accuracy of DMFBTSM using Riemannian distance is 1.1% higher than that of MFBTSM, the training time is shortened by 1.7 times, and the test time is shortened by 1.7 times. The test time and training time of DMFBTSM with three time windows are approximately equal to those of MFBTSM with one time window, but the classification accuracy is improved by 2.56%. The performance is improved, mainly because DMFBTSM eliminates the poorly separable frequency bands in the MI task of the subject, making the extracted features more effective and reducing the dimensionality of the feature vector. As a result, the probability of overfitting of the classifier due to much high dimension of the feature vectors in the case of limited samples will decrease.

In addition, the average classification accuracy of DMFBTSM using Riemannian distance is higher than that of DMFBTSM using Euclidean distance, and the test time is close to the training time. In the case of three time windows (T1, T2, and T3) and one time window (T1), the classification accuracy of DMFBTSM using Riemannian distance is 0.8 and 1.49% higher than that of DMFBTSM using Euclidean distance. It should be noted that not every subject’s MI classification accuracy will be improved due to the choice of frequency band. For subject A4, the classification accuracy of DMFBTSM is lower than that of MFBTSM. The performance is improved, mainly because DMFBTSM eliminates the poorly separable frequency bands in the MI task of the subject, making the extracted features more effective and reducing the dimensionality of the feature vectors, so that the classifier would not overfit due to the too high dimension of the feature vectors in the case of limited samples.

**FIGURE 6:**
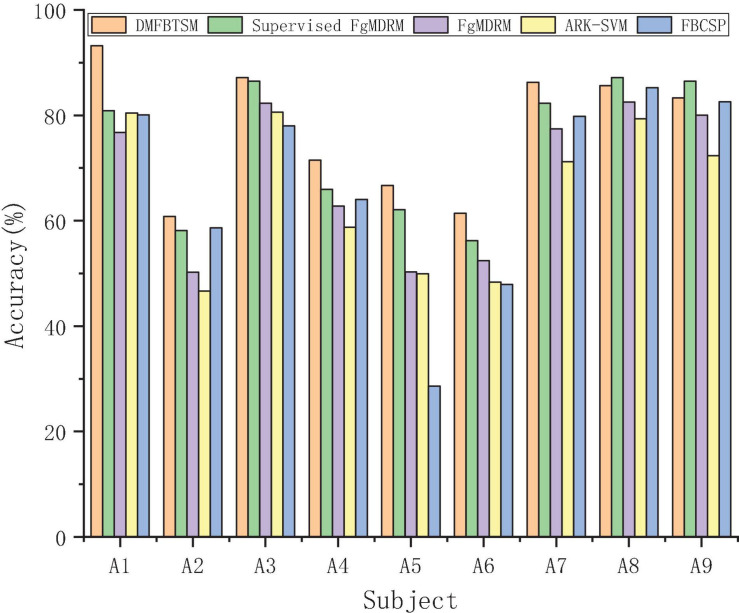
According to different related methods, classification accuracy is compared on the test set (Session 2) of 9 subjects in data-set 2a.

It can be seen from Table 2 that the average classification accuracy of Supervised FgMDRM is 5.66% higher than that of FgMDRM. This is because the combination of FgMDRM and the RETRAIN adaptive strategy allows the classifier to add new samples during the testing session and continuously retrain. However, the retraining process is supervised and requires the real labels of the new samples. In addition, the role of this adaptive technology is related to the subjects’ proficiency in BCI, because the more proficient the subjects, the more stable EEG patterns are produced., So that more effective samples can be used for retraining. The average accuracy of DMFBTSM is approximately 12% higher than that of ARK-SVM, which shows that DMFBTSM can extract more sufficient, more robust and more robust Riemann covariance features than single-time band TSM. The average classification accuracy of DMFBTSM with the best result is 3.36% higher than that of the supervised FgMDRM with the second best result, and it can be seen from Figure 6 that except for the two subjects A8 and A9, PMFBTSM achieved the best results among other subjects. This result is also reasonable. The TSM-based Riemann method can use techniques such as filter bank analysis and band selection to extract more effective features and combine the advantages of the chosen classifier to generate more complex decision functions. Although TSM-based Riemann methods have better overall function than MDRM methods, they are not suitable for online operation because of the increased algorithmic complexity and possible need of intense learning inherited by the classifier. The average accuracy of DMFBTSM is approximately 10% higher than that of FBCSP, which is the classical method of frequency domain feature extraction using filter bank analysis and spatial filtering. The results are compared to better evaluate the proposed method.

As can be seen from Table 3, the average accuracy of the proposed method through 10-fold cross-validation on the test set is 9.23% and 8.02% higher than the two classical deep learning methods Shallow-ConvNet and CW-ConvNet, respectively, 2.58% higher than the latest deep learning method the Deep multi-view feature learning, and 2.68 and 4.65% higher than that of the Monolithic Network and DFFN methods, respectively. The first Deep Learning method proposes a new deep multi-view feature learning method in order to obtain more representative moving image features from EEG signals. The last three Deep Learning algorithms adopted ConvNet to learn the spatial characteristics extracted by CSP (Xu et al., 2020). Compared with the traditional CSP method, the Riemann geometric method based on TSM takes into account the nonlinear information contained in the covariance matrix, and can extract more abundant and effective features. Moreover, the method is an unsupervised operation, which can reduce the time of feature extraction (Congedo et al., 2017). These Deep Learning-based methods mentioned above are very useful, and have their own advantages and disadvantages and their respective suitable occasions compared with the methods mentioned in this article. As highlighted in Yger et al. (2017), the processing procedures of Riemannian approaches such as MDRM is simpler and involves fewer stages than more classic approaches. Also, Riemannian classifiers apply equally well to all BCI paradigms (e.g., BCIs based on mental imagery, ERPs and SSVEP); only the manner in which data points are mapped in the SPD manifold differs (Congedo et al., 2017). Another disadvantage of the Riemann method is that the TSM-based method seems to increase the number of sensors (so the greater the dimension of the covariance matrix), the worse the classification accuracy will become (Yger et al., 2017). This may be due to the fact that the increase in the transformation dimension requires more attention. When almost singular covariance matrices are generated, they cannot be effectively processed by Riemannian geometry (Yger et al., 2015).

In our future work, we will try to combine some new Deep Learning classifiers with DMFBTSM method to further improve the classification accuracy of multi-class MI-BCI. In addition, the methods proposed in this article will extract a large number of real-valued Riemannian covariance features, thus increasing the number of weights and the complexity of classifiers, which makes them unsuitable for real-time execution on devices with limited resources. Therefore, it is considered to combine regularization, sparse feature selection and other techniques with linear classification to deal with a large number of Riemannian covariance features, so that the model obtained by training will have less memory footprint and better classification performance.

## Conclusion

A Discriminative and multi-scale Filter Bank Tangent Space Mapping (DMFBTSM) algorithm is proposed in this article to design the FB of a specific subject. On the 4-class BCI competition IV-2a data set, the average classification accuracy of nine subjects reached 77.33 ± 12.3%. When the training time and the test time are similar, the classification accuracy is increased by 2.56% compared to MFBTSM. When the classification accuracy is similar, the training speed is increased by more than three times, and the test speed is increased two times more. Compared with Supervised Fisher Geodesic Minimum Distance to the Mean (Supervised FGMDRM), another new variant based on Riemann geometry classifier, the average accuracy is 3.36% higher. The results show that the proposed DMFBTSM algorithm can be expected to select a frequency band with good separability for specific subjects to improve the classification accuracy of multiclass MI tasks.

Our future work is to apply the proposed method to neurofeedback to further improve the classification accuracy of multi-class MI-BCI.

## Data Availability Statement

Publicly available datasets were analyzed in this study. This data can be found here: http://bbci.de/competition/.

## Ethics Statement

The studies involving human participants were reviewed and approved by the Medical Ethics Committee of Kunming University of Science and Technology School of Medicine. The patients/participants provided their written informed consent to participate in this study.

## Author Contributions

FW: conceptualization, methodology, programming, and writing and editing. AG: methodology, writing – reviewing and editing. LZ: designing the experiment. WZ: investigation and validation. HL: investigation and checking language. YF: perfecting the model and revising the manuscript, project administration, funding acquisition, and supervision. All authors contributed to the article and approved the submitted version.

## Conflict of Interest

The authors declare that the research was conducted in the absence of any commercial or financial relationships that could be construed as a potential conflict of interest.

## Footnotes

1 http://bbci.de/competition/
